# Flagellin shifts 3D bronchospheres towards mucus hyperproduction

**DOI:** 10.1186/s12931-020-01486-x

**Published:** 2020-08-26

**Authors:** Richard F. Sprott, Felix Ritzmann, Frank Langer, Yiwen Yao, Christian Herr, Yvonne Kohl, Thomas Tschernig, Robert Bals, Christoph Beisswenger

**Affiliations:** 1 Department of Internal Medicine V – Pulmonology, Allergology and Respiratory Critical Care Medicine, Saarland University, Kirrberger Str. 100, Building 41M, 66421 Homburg/Saar, Germany; 2 Department of Thoracic and Cardiovascular Surgery, Saarland University, 66421 Homburg, Germany; 3 Department Bioprocessing & Bioanalytics, Fraunhofer Institute for Biomedical Engineering, Joseph-von-Fraunhofer-Weg 1, 66280 Sulzbach, Germany; 4 Institute of Anatomy and Cell Biology, Saarland University, 66421 Homburg, Germany

**Keywords:** Organoids, Bronchospheres, Mucus, PAMPs, Flagellin

## Abstract

Cystic fibrosis (CF) and chronic obstructive pulmonary disease (COPD) are associated with acute and chronic bacterial infections of the lung. Excessive differentiation of basal cells to mucus-producing goblet cells can result in mucus hyperproduction and loss of mucociliary clearance in the airways of CF and COPD patients. Here, we aimed to investigate the effect of pathogen-associated molecular patterns (PAMPs) on the differentiation of human 3D bronchospheres. Primary human bronchial epithelial cells (HBECs) were differentiated to bronchospheres in the presence of bacterial flagellin and LPS and the synthetic Toll-like receptor (TLR) ligands Pam3CSK4 (TLR-2) and polyinosinic:polycytidylic acid (pIC, TLR-3). Electron and fluorescence microscopy showed that the differentiation of bronchospheres associated with the formation of lumina and appearance of cilia within 30 days after seeding. Incubation with flagellin resulted in a decreased formation of lumina and loss of cilia formation. Incubation with Pam3CSK, pIC, and LPS did not significantly affect formation of lumina and ciliation. Mucus production was strongly increased in response to flagellin and, to a lesser degree, in response to Pam3CSK4. Our results indicate that bacterial factors, such as flagellin, drive the differentiation of the respiratory epithelium towards mucus hyperproduction.

## Introduction

Airway remodeling contributes to air flow limitation and loss of lung structure in chronic respiratory diseases, such as CF and COPD. In both diseases, basal and goblet cell hyperplasia leads to mucus hyperproduction, loss of mucociliary clearance, disturbed epithelial barrier function, and impaired regeneration of the airway epithelium [1, 2]. Therefore, the respiratory epithelium is a potential target for therapeutic intervention addressing basal cell differentiation and mucus hyperproduction [1].

CF patients suffer from persistent bacterial infections of the lung with *Pseudomonas aeruginosa* being a dominant pathogen later in life [3]. In COPD, nontypeable *Haemophilus influenzae* (NTHi) contributes to pulmonary inflammation and acute exacerbations [4]. Infections and colonization in the lung with these pathogens associate with a constant contact of the airway epithelium with PAMPs (e.g. lipopeptide, LPS, flagellin) which activate innate immunity responses via pattern recognition receptors (PRRs), such as TLRs. Even though the initiation of innate immunity is required for an adequate host response, prolonged and excessive activation of innate inflammatory responses may be harmful and therefore contribute to the progression of CF and COPD [3–6].

In recent years, 3D organoid models have been established to study tissue differentiation and therapeutic interventions. Pre-clinical studies suggest that transplanted primary lung organoids can be used for cell-based therapies for the treatment of chronic lung diseases, such as COPD or IPF [7–9]. Importantly, 3D organoid models have the potential to reduce and replace animal studies. A recent manuscript showed that exposure of developing bronchospheres to inflammatory mediators (e.g. IL-13) drives goblet cell metaplasia [10]. The purpose of this study was to investigate the consequences of the exposure to PAMPs on the differentiation of human 3D bronchospheres. We demonstrate that bacterial-derived factors, such as flagellin, shift 3D bronchospheres towards mucus hyperproduction. Moreover, exposure to flagellin resulted in a loss of ciliation.

## Methods

HBECs were isolated from patients who underwent surgery because of lung cancer as described previously [11]. Only cancer-free tissue was used for cell isolation. The protocol was approved by the institutional review board (ethics committee) of the Landesärztekammer of the State of Saarland, and informed consent was obtained from the patients. Organoids were cultured as described before [10]. In brief, 80 μl of passage 1 cells (3 × 10^4^ cells per ml differentiation media containing 5% Matrigel) were plated in each well of a 96-well plate containing 40 μl per well of a 25% Matrigel (Corning, USA) solution as base layer. 120 μl differentiation media was added at day 2 containing 100 ng/ml of Pam3CSK4, polyinosinic:polycytidylic acid (pIC), ultrapure LPS from *Escherichia coli* 055:B5, or flagellin from *P. aeruginosa* (Invivogen, USA) and 5% Matrigel. Media was changed every 4 days. The presence of lumina, cilia beating, and diameter were determined under the phase contrast microscope 14, 22, and 30 days after seeding. Organoids from each condition were pooled and embedded in paraffin 30 days after seeding. Paraffin sections (2 μM thickness) were stained with haematoxilin-eosin (H&E). Deparaffinized paraffin sections were treated with BSA (1%) and Tween-20 in PBS (0.1%) and incubated overnight at 4 °C with the primary antibodies for MUC5B (sc-393,952, Santa Cruz, USA, 1/100) and KRT5 (ab-75,869, Abcam, USA, 1/100) in PBS containing BSA (1%). Cells were incubated for 30 min with secondary antibodies (goat anti-mouse FITC, Sigma, Germany; goat anti-rabbit, Cyanine 5, Thermo-Fisher Scientific, Germany). Immunohistochemistry for MUC5AC (ab-3649, Abcam) was performed as described before [12]. To analyze and merge images, we used ImageJ software (National Institutes of Health). Western blot was performed as described before [12]. Membranes were probed with monoclonal antibodies directed against MUC5B (sc-393,952, Santa Cruz) and β-actin (#4967, Cell Signaling Technology, USA). The ultrastructure of untreated bronchospheres were analyzed by electron microscopy as described before [13]. Ultra-thin sections were contrasted with uranylacetate using routine procedures. The analysis was performed using a FEI Technai 12 transmission electron microscope (FEI, Thermo-Fisher Scientific) at 100 kV, equipped with a digital 8-bit camera. Comparisons between groups were analyzed by t test (two-sided) using the software Prism (GraphPad Software, USA). Technical replicates were combined to a single data point for each independent experiment. The results were considered statistically significant for *P* < 0.05.

## Results

**Fig. 1:**
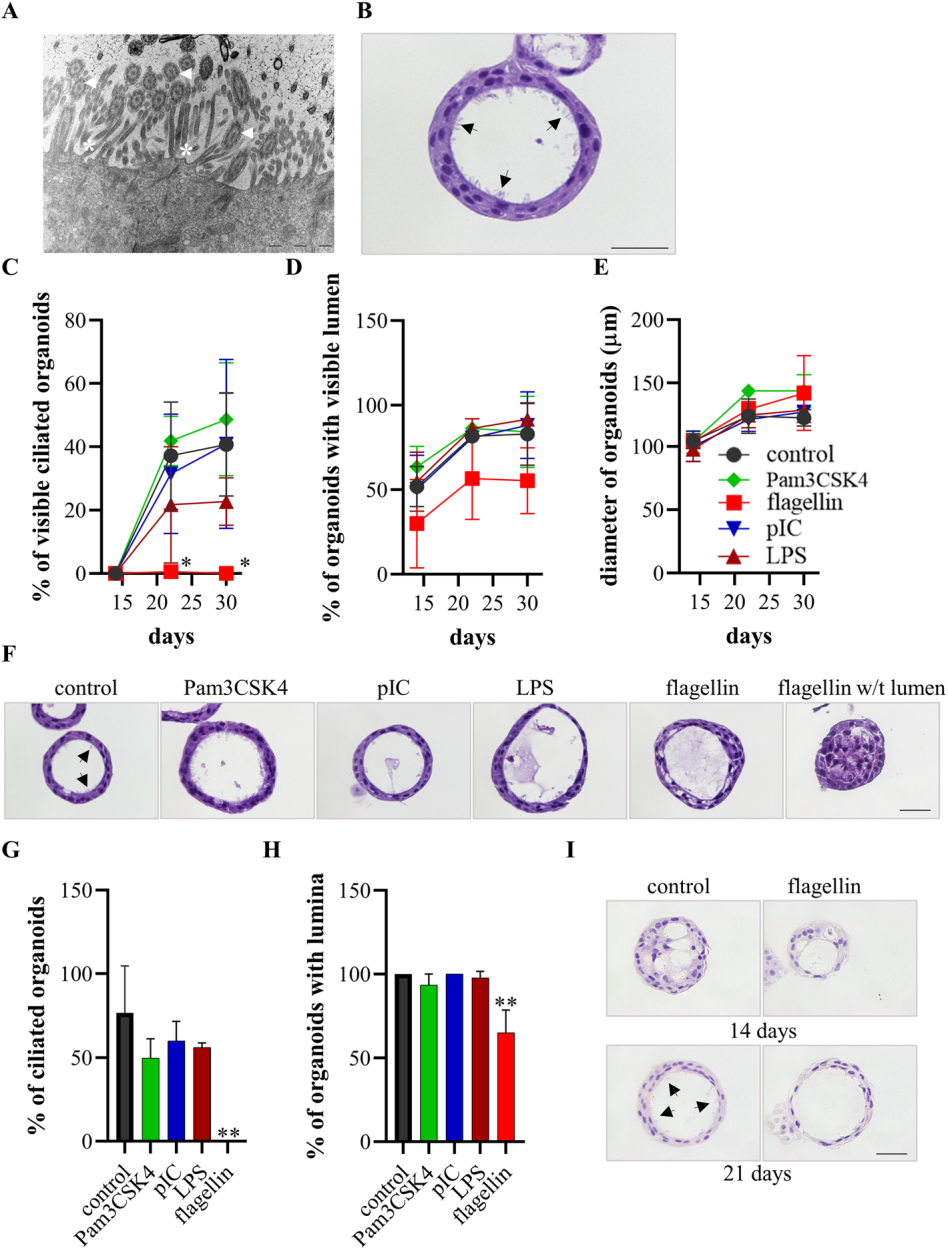
Flagellin inhibits the formation of cilia. **a** The ultrastructure of the inner surface of a bronchosphere embedded 30 days after seeding (23.000-fold magnification). Arrows indicate kinocilia and * indicate microvilli. **b** Representative H&E staining of a bronchosphere embedded 30 days after seeding. Scale bar: 20 μm. Arrows indicate cilia. (**c** to **f**) Developing bronchospheres were treated with different PAMPs during the differentiation phase starting at day 2 after seeding. Cilia beating (**c**), the presence of lumina (**d**), and diameter (**e)** were determined under the phase contrast microscope 14, 22, and 30 days after seeding. (**f**) Representative H&E staining of bronchosphere cultured under the indicated conditions embedded 30 days after seeding. Arrows indicate cilia. Scale bar: 20 μm. The percentage of bronchospheres with cilia (**g**) and lumina (**h**) were analyzed H&E-stained sections. Data were compared by unpaired Student’s t-test and are shown as the mean ± SEM from three independent experiments. *p < 0.05, **p < 0.01. (**i**) H&E staining of bronchospheres cultured under the indicated conditions embedded 14 and 21 days after seeding. Arrows indicate cilia. Scale bar: 20 μm

Transmission electron microscopy and H&E staining showed that HBECs seeded in matrigel formed differentiated bronchospheres with microvilli and typical kinocilia on its surface (Fig. 1a and b). We studied whether PAMPs modulate the differentiation of bronchospheres. Therefore, we treated the developing bronchospheres with different ligands for TLRs (Pam3CSK4, pIC, LPS, flagellin) during the differentiation phase starting at day 2 after seeding. Examination under the phase contrast microscope showed that the differentiation of bronchospheres associated with the formation of lumina preceding visible cilia beating within 30 days after seeding (Fig. 1c to e). Treatment with flagellin resulted in an almost complete loss of visible cilia beating, but did not significantly affect formation of lumina and the diameter of the bronchospheres. Treatment with Pam3CSK4, pIC, and LPS did not significantly affect formation of lumina and ciliation. H&E staining of bronchospheres embedded 30 days after seeding confirmed that treatment with flagellin but not Pam3CSK4, pIC, and LPS inhibited the formation of cilia (Fig. 1f). Ciliated cells were identified in approximately 75% of control bronchospheres, whereas no ciliated cells were found in bronchospheres treated with flagellin (Fig. 1g). Moreover, the percentage of organoids with a lumen was significantly reduced in the flagellin-treated group (Fig. 1h). H&E staining of paraffin embedded bronchospheres confirmed the presence of cilia in control bronchospheres, but not in the flagellin treated group 21 days after seeding (Fig. 1i).

**Fig. 2:**
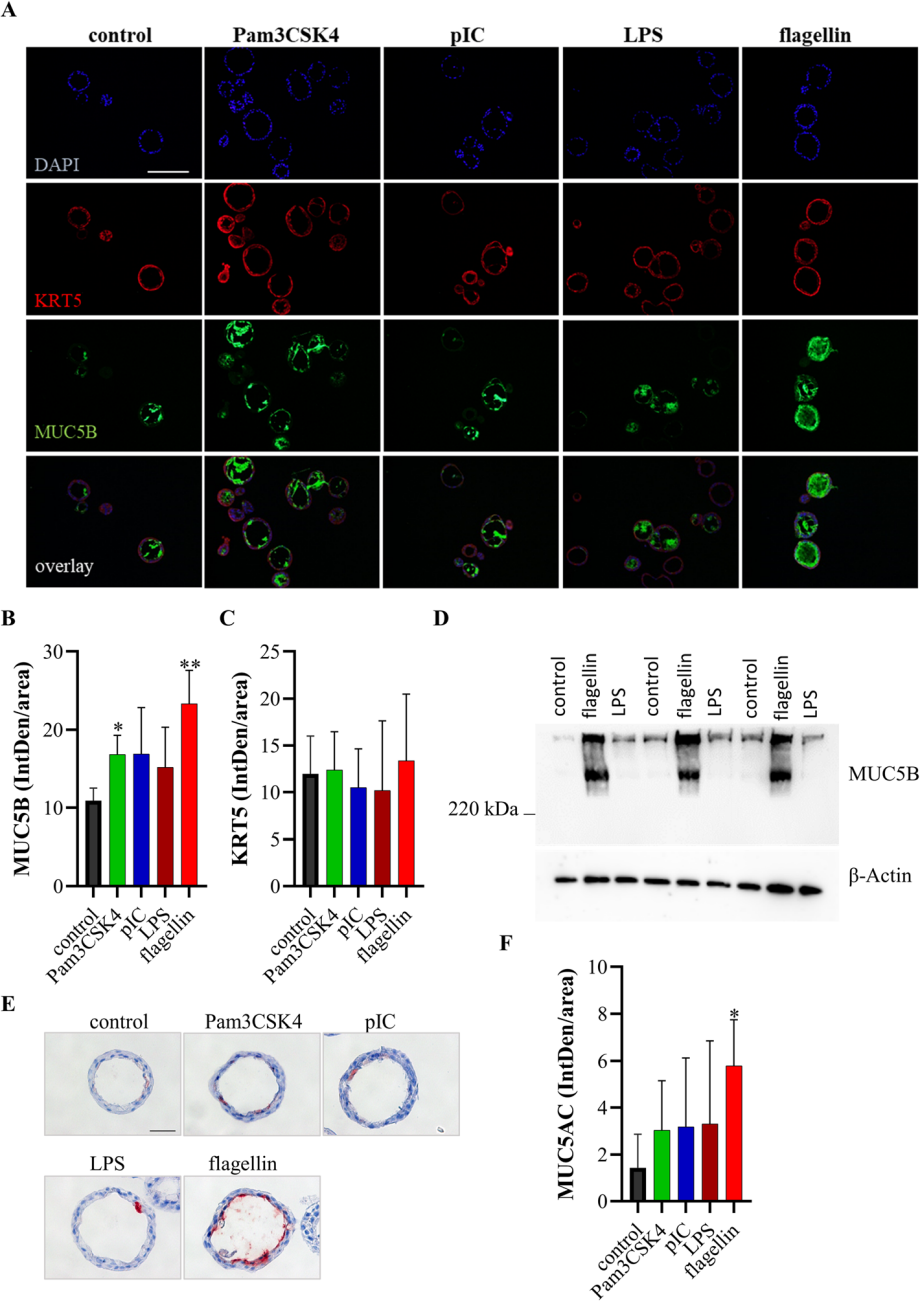
Flagellin induces mucus secretion. Developing bronchospheres were treated with different PAMPs during the differentiation phase starting at day 2 after seeding. Bronchospheres were analyzed 30 days after seeding. **a** Immunofluorescence staining was performed for KRT5 (red channel) and MUC5B (green channel). Nuclei were stained with DAPI (blue). Scale bar: 100 μm. **b** Quantification of MUC5B staining. **c** Quantification of KRT5 staining. **d** Immunoblot analysis of lysates of bronchospheres cultured in the presence of flagellin, LPS, or control media probed with antibody to MUC5B or β-actin. **e** Immunohistochemistry was performed for MUC5AC. Scale bar: 20 μm. **f** Quantification of MUC5AC staining. Data were compared by unpaired Student’s t-test and are shown as the mean ± SEM from three independent experiments. *p < 0.05, **p < 0.01

To study whether the different conditions affected mucus production we stained bronchospheres for the goblet cell marker mucin 5B (MUC5B) and cytokeratin 5 (KRT5), a marker for basal cells, 30 days after seeding (Fig. 2a). Quantification of the staining showed that treatment with flagellin resulted in a strongly increased expression of MUC5B (Fig. 2b), whereas there was no significant difference in the expression of KRT5 (Fig. 2c). Treatment with Pam3CSK also resulted in a significantly increased expression of MUC5B. In addition, western blot analysis demonstrated an increased expression of MUC5B in flagellin-treated bronchospheres 30 days after seeding of the basal cells (Fig. 2d). Immunohistochemistry further showed that treatment with flagellin also resulted in an increased expression of the mucin MUC5AC (Fig. 2 e and f).

## Discussion

CF and COPD are characterized by chronic bacterial colonization of the airways [3, 4]. Therefore, the airway epithelium of CF and COPD patients is constantly exposed to bacterial components (e.g. flagellin, LPS, lipopeptides). In the present study, we show by using an in vitro organoid model for the differentiation of mucociliary epithelium from basal cells [10] that exposure to flagellin results in mucus hyperproduction and loss of ciliation, whereas exposure to the synthetic lipopeptide Pam3CSK only increased mucus production. These results suggest that prolonged activation of innate immune mechanisms by bacteria via PRRs such as TLR-5 drives the differentiation of basal cells towards mucus-producing goblet cells and suppresses formation of cilia. Thus, the interaction of bacterial components with respiratory cells may promote basal and goblet cell metaplasia and hinder mucociliary clearance as well as the regeneration of injured epithelium and tissue homeostasis in the airways of CF and COPD patients.

*P. aeruginosa*-derived flagellin had a strong effect on the differentiation of the mucociliary epithelium. Studies showed that HBECs cells detect *P. aeruginosa* via recognition of flagellin by TLR-5 and that approximately 75% of clinical isolates of *P. aeruginosa* retain TLR5 activating capacity during chronic CF lung infection [14, 15]. However, as cytosolic receptors (e.g. NLRC4 inflammasome receptors) also detect flagellin [16] we cannot exclude signaling pathways independent of TLR-5 contributing to the flagellin-induced mucus hyperproduction. In any case, our data indicate that bacterial components can cause structural changes in the airways of CF patients independent of CFTR (Cystic Fibrosis Transmembrane Conductance Regulator). This assumption is support by findings of Adam et al. showing that air liquid interface cultures (ALI) with freshly isolated cells obtained from CF patients have a hyperinflammatory phenotype and exhibit basal cell hyperplasia without increased numbers of goblet cells as compared to cultures with cells obtained from non-CF patients. However, ALI cultures with passages 1 CF cells did not show a hyperinflammatory phenotype anymore, nor did they longer reconstitute a remodeled epithelium. Moreover, a mixture of pro-inflammatory cytokines increased numbers of goblet cells independent of mutations in CFTR [17]. Thus, goblet metaplasia and mucus hyperproduction may relate to bacterial infections and inflammation rather than to CFTR dysfunction [1, 17].

Danahay et al. showed that incubation of developing bronchospheres with interleukin (IL)-13 and IL-17A, a cytokine that is suggested to mediate *P. aeruginosa*-induced lung inflammation [18, 19], results in increased mucin production and decreased expression of markers of ciliated cells in an Notch2-dependent manner [10]. Our data indicate that direct activation of basal cells by bacteria also promotes goblet cell metaplasia and airway remodeling in chronic respiratory diseases. Additional studies are needed to clarify whether there are specific signaling pathways that regulate cell differentiation in response to cytokines and bacteria and whether cytokines and bacteria synergistically induce goblet cell metaplasia.

In summary, our data indicate that ongoing activation of innate immune pathways in basal cells promotes goblet cell metaplasia and airway remodeling as seen in chronic respiratory diseases such as CF and COPD. Thus, 3D bronchosphere models are useful tools for the identification of druggable signaling pathways that mediate mucus hyperproduction and loss of ciliation, thereby reducing and replacing animal studies. As therapeutic interventions targeting basal cell differentiation and mucus hyperproduction have the potential to improve lung function in CF and COPD one approach could be the inhibition of TLR, inflammasome, or Notch signaling pathways using small molecules or antibodies in 3D bronchospheres models [15, 20].

## Data Availability

The datasets generated and analyzed during the current study are available from the corresponding author on reasonable request.

## Abbreviations

CF: Cystic fibrosis
CFTR: Cystic fibrosis transmembrane conductance regulator
COPD: Chronic obstructive pulmonary disease
IPF: Idiopathic pulmonary fibrosis
PAMPs: Pathogen-associated molecular patterns
TLR: Toll-like receptor
pIC: Polyinosinic:polycytidylic acid
HBECs: Human bronchial epithelial cells
NTHi: Nontypeable *Haemophilus influenzae*
Muc5B: Mucin 5B
KRT5: Cytokeratin 5
ALI: Air liquid interface
